# Benzyl isothiocyanate as an alternative to antibiotics? a comparative *in vivo* study using *Pseudomonas aeruginosa* infection as a model

**DOI:** 10.1371/journal.pone.0303490

**Published:** 2024-05-16

**Authors:** Jian Yang, Tousif Ahmed Hediyal, Saravana Babu Chidambaram, Ruchika Kaul-Ghanekar, Meena Kishore Sakharkar

**Affiliations:** 1 College of Pharmacy and Nutrition, University of Saskatchewan, Saskatoon, SK, Canada; 2 Department of Pharmacology, JSS College of Pharmacy, JSS Academy of Higher Education & Research, Mysuru, Karnataka, India; 3 Center for Experimental Pharmacology and Toxicology, JSS Academy of Higher Education & Research, Mysuru, Karnataka, India; 4 Cancer Research Lab, Symbiosis School of Biological Sciences (SSBS), Symbiosis Centre for Research and Innovation (SCRI), Symbiosis International (Deemed University), Pune, Maharashtra, India; University of Ibadan Faculty of Science, NIGERIA

## Abstract

Due to over-prescription of antibiotics, antimicrobial resistance has emerged to be a critical concern globally. Many countries have tightened the control of antibiotic usage, which, in turn, promotes the search for alternatives to antibiotics. Quite a few phytochemicals have been investigated. Benzyl isothiocyanate (BITC) is an important secondary metabolite in cruciferous species and exhibited potent antimicrobial activity under *in vitro* conditions. In this research, we undertook a comparative mouse model study of BITC with gentamycin sulfate (positive antibiotic control) and ceftiofur hydrochloride (negative antibiotic control) against *Pseudomonas aeruginosa* infection. Our results showed that BITC exhibited comparable or better antimicrobial activity and lower infiltration of mouse immune cells upon comparing to gentamycin sulfate. Furthermore, BITC did not impose any toxicity to the air pouch skin tissues. In summary, our current study suggests that BITC could be an alternative to antibiotics and deserves further *in vivo* and clinical trial studies.

## Introduction

Despite warnings of overuse from various governing agencies such as the World Health Organization (WHO), antibiotics have been overprescribed worldwide, which, in turn, promotes the rapid development of antimicrobial resistances and transfer of bacteria and genes across different hosts [1]. To combat antimicrobial resistance, many countries have tightened the control of antibiotic usage. However, this tight control has prompted the search for alternatives to antibiotics; and quite a few phytochemicals have been investigated for their antimicrobial activities [2,3]. For any phytochemical to be an alternative to antibiotics, it should minimally fulfill two requirements. First, the phytochemical should possess comparable antimicrobial activities to antibiotics; and secondly, it exhibits minimal toxicity to the hosts.

Benzyl isothiocyanate (BITC) is an important secondary metabolite widely present in cruciferous species such as broccoli, cabbage, and radish. It possesses a wide range of biological functions, including antibacterial, anti-inflammatory, and anticancer activities [4–7]. BITC has been shown to be a potent broad-spectrum antibacterial agent, and its acute LD_50_ (median lethal dose) was determined to be 400 mg/kg for 24 hr and 350 mg/kg for 48 hr, respectively, in male Swiss Webster mice [4,7]. However, most of the antibacterial studies on BITC were undertaken under *in vitro* conditions and without positive and negative antibiotic controls even for the *in vivo* studies [4,7–10]. Therefore, to evaluate whether BITC could be applied as an alternative to antibiotics, we initiated a comparative *in vivo* study on the antibacterial activity of BITC against *Pseudomonas aeruginosa* infection using gentamycin sulfate (GS) as a positive antibiotic control and ceftiofur hydrochloride (CH, veterinary usage) as a negative antibiotic control, respectively. Our results showed that BITC exhibited a better pharmacological function than GS and is a promising alternative candidate to antibiotics.

## Results and discussion

### Mortality observation

No treatment-related mortality or moribund state was observed in the experimental animals throughout the study period.

### Effects of BITC on bacterial load

**Fig 1:**
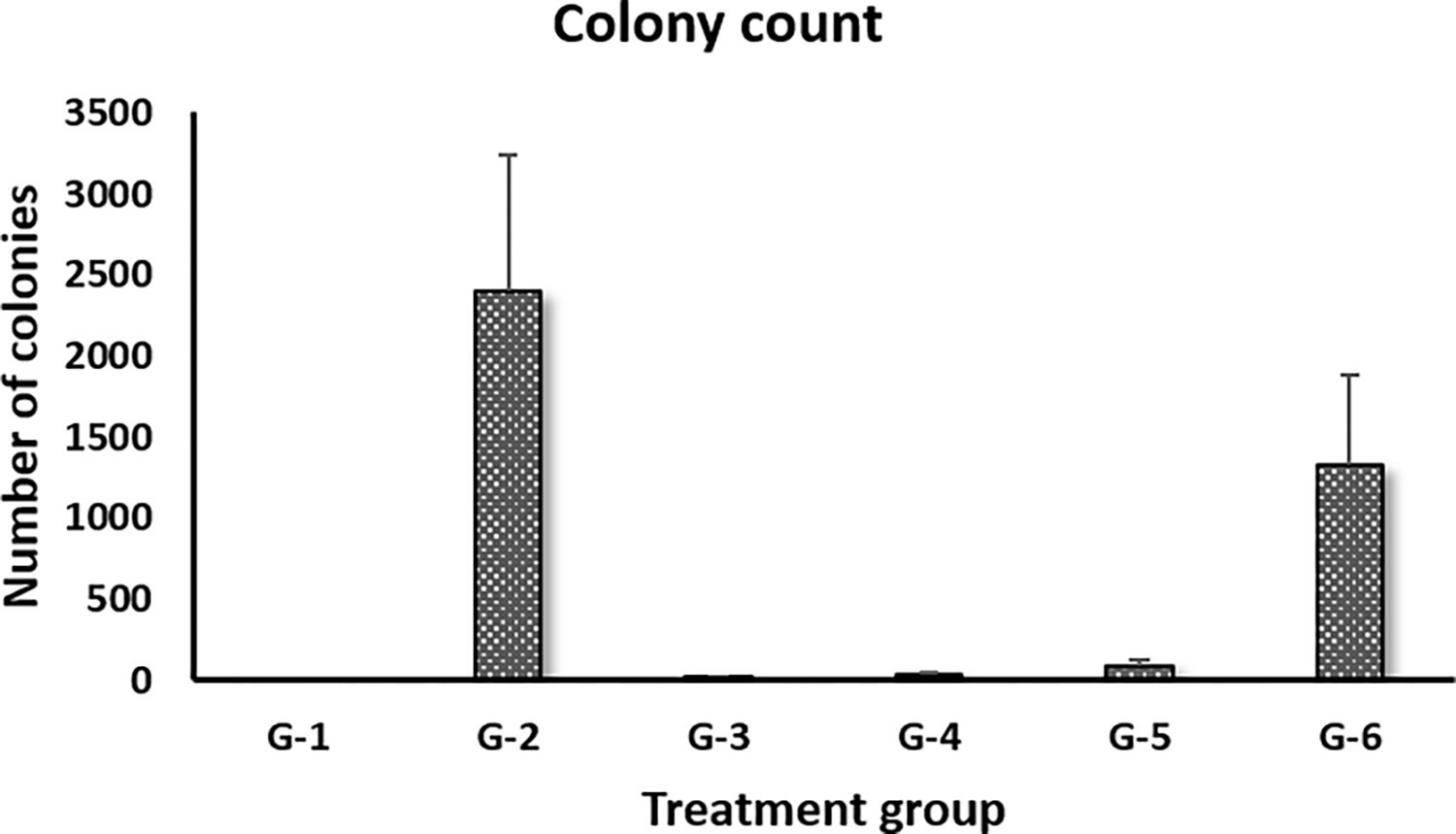
Bacterial load in air pouch fluid of each group after 7 days of treatment. Group information was provided in Table 1.

Seven days after treatment, three mice from each group were randomly selected and anaesthetized using ketamine-xylazine. Subsequently, approximately 1–2 mL exudate was aspired from each air pouch. The air pouch exudate contained both bacterial cells and infiltrated mouse immune cells. Bacterial load was determined by plating the air pouch liquid on nutrient agar plates and counting the number of colonies formed after culture. As shown in Fig 1, the positive control of infection G-2 group has the highest bacterial load of 2396 ± 836 colonies. High bacterial load was also observed for the negative antibiotic control G-6 group, which is consistent with previous studies that *P*. *aeruginosa* is resistant towards ceftiofur [11,12]. It was about 55.4% of the count recorded for G-2. However, the low-dose BITC treatment G-3 group, high-dose BITC treatment G-4 group, and positive antibiotic control G-5 group showed only marginal bacterial loads, which were approximately 0.8%, 1.4% and 3.4%, respectively, of the G-2 positive control. This suggests that BITC possessed potent inhibitory activity against *P*. *aeruginosa* and exhibited similar antimicrobial efficacy as gentamycin sulfate, the positive antibiotic control. Furthermore, the doses of BITC (25 mg/kg and 50 mg/kg) were significantly lower than the *in vitro* MIC (minimum inhibitory concentration) of 2145±249 μg/mL against different *P*. *aeruginosa* isolates [13], implicating that BITC is likely to exert a much better antimicrobial efficacy in the presence of immune system.

### Effects of BITC on immune cell inflitration

**Fig 2:**
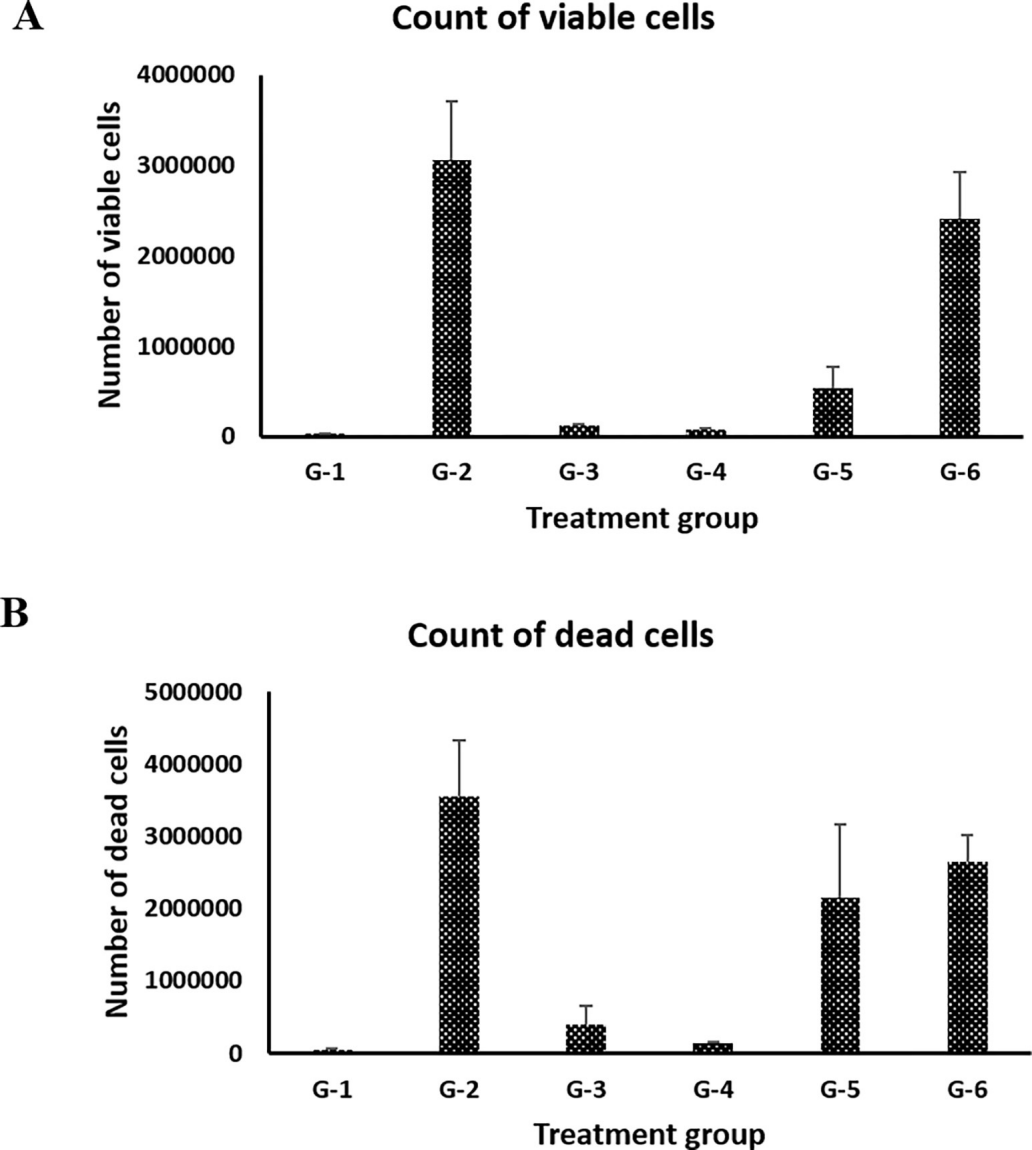
Count of viable infiltrated immune cells (**A**) and count of dead infiltrated immune cells (**B**) in the air pouch fluid of the six treatment groups.

Immune cell infiltration is a normal process upon bacterial infection and reflects the interaction between the host and bacterial cells. To get an understanding of mouse immune cell filtration in each treatment group, viable and dead cells in the air pouch fluids were determined using a previously published protocol [14,15]. The number of dead cells was derived by subtracting the number of viable cells from the number of total cells. As illustrated in Fig 2, the positive control of infection G-2 group has the highest number of infiltrated cells (46.3% viable and 53.7% dead). We also observed a small number of infiltrated cells (41.0% viable and 59% dead) in the negative control of infection G-1 group. It was only about 1.3% of the G-2 group. This small amount of immune cell infiltration was likely due to air pouch formation and may be regarded as the background information of air pouch for all treatment groups. As expected, the negative antibiotic control G-6 group had a large number of infiltrated cells (47.7% viable and 52.3% dead), which was about 76.4% of the G-2 group. This implies that reduced bacterial load of G-6 group (55.2% of positive control) was predominately caused by the host immune responses. In the positive antibiotic control G-5 group, the number of infiltrated cells was approximately 40.5% of the positive control G-2 group, with 20.1% viable and 79.9% dead.

For the two BITC treatment groups, only a small number of infiltrated cells were detected. The numbers of infiltrated cells in the G-3 group (24.1% viable and 75.9% dead) and G-4 group (37.5% viable and 62.5% dead) were approximately 7.7% and 3.1% of the G-2 group, respectively. Because BITC possesses potent anti-inflammatory activity [16,17], it can decrease the infiltration of immune cells like monocytes and macrophages. However, despite the much lower level of immune cell infiltration, BITC exhibited a comparable antimicrobial effect as gentamycin sulfate and almost completely suppressed the growth of *P*. *aeruginosa*. This implicates that BITC may be more potent than gentamycin sulfate under the current experimental condition. Furthermore, it is noteworthy to point out that cell viability of the infiltrated immune cells was less than 50% in all treatment groups. We hypothesize that *P*. *aeruginosa* can kill host immune cells via the type III secretion system (T3SS) proteins, which has been previously reported to be involved in immune response modulation upon bacterial infection [18–20]. Further studies are warranted to prove the hypothesis by examining the expression of T3SS proteins and persister cell population of *P*. *aeruginosa*.

### Effects of BITC to local air-pouch skin tissues

**Fig 3:**
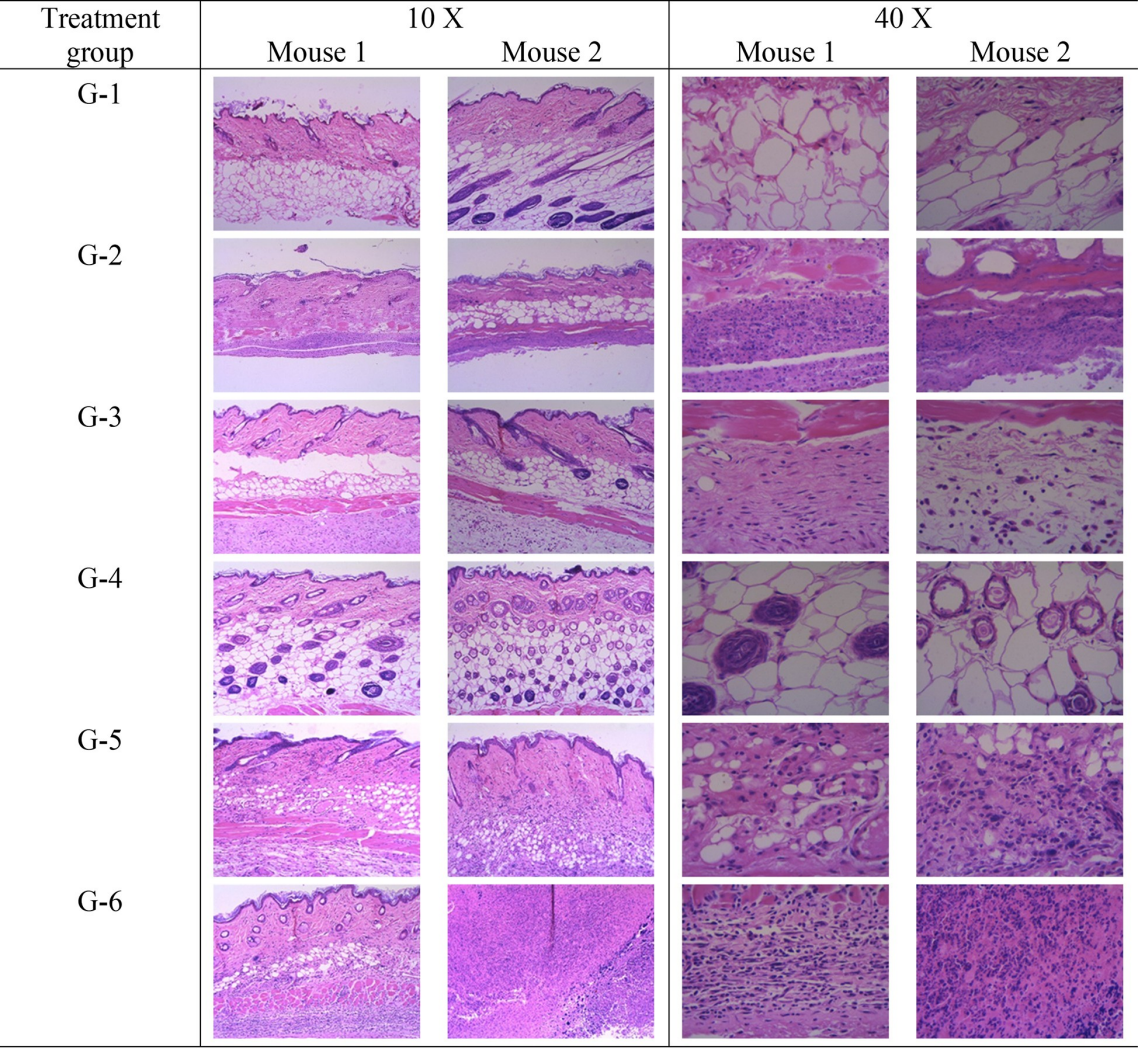
Staining of the air pouch skin tissues by hematoxylin and eosin (H&E) stain. For each treatment group, skin tissues were collected from two randomly selected mice. Images were taken under both 10 X and 40 X magnification fields.

We finally examined whether BITC would elicit local toxicity to the air pouch skin tissues, although the two administered doses were significantly lower than its acute LD_50_ values. After 7 days of treatment, two mice from each group were randomly selected and euthanized. Then, skin tissues were collected from the air pouches, processed to have section thickness of 5–6 μm, and stained by hematoxylin and eosin (H&E) stain. As shown in Table 1 and Fig 3, no inflammation was observed in the negative control of infection G-1 group. However, in the positive control of infection G-2 group, inoculation of *P*. *aeruginosa* induced moderate inflammation. Skin tissue inflammation was reduced to minimum in the G-3 group, which was treated with low dose of BITC (25 mg/kg), and diminished in the G-4 group, which was treated with high dose of BITC (50 mg/kg). These results are consistent with previous observation that BITC possesses potent antimicrobial, anti-inflammatory and antioxidant activities [4,7,16,17,21]. In the positive antibiotic control G-5 group, the mice experienced minimum to moderate inflammation in the air pouches; whereas in the negative antibiotic control G-6 group, both mice experienced severe inflammation in their air pouches. Thus, our current study suggests that BITC can significantly reduce local skin inflammation induced by *P*. *aeruginosa* inoculation and may provide a better protection of the host than the gentamycin sulfate, the positive antibiotic control.

**Table 1:** Histopathological analysis of mouse air pouch skin tissues with the grading system as following, 0: Within normal limit; 1: Minimal inflammation; 2: Slight inflammation; 3: Moderate inflammation; and 4: Severe inflammation.

Group	Mice	Grade of inflammation	Mean of grades
G-1	No. 1	0	0
	No. 2	0	
G-2	No. 1	3	3
	No. 2	3	
G-3	No. 1	1	1
	No. 2	1	
G-4	No. 1	0	0
	No. 2	0	
G-5	No. 1	1	2
	No. 2	3	
G-6	No. 1	4	4
	No. 2	4	

## Materials and methods

### Preparation of *P*. *aeruginosa* inoculum

*P*. *aeruginosa* ATCC 27853 was cultivated on a Nutrient Agar plate at 37°C overnight and then sub-cultured in Nutrient Broth at 80–90°C for 24 hr. The cultured bacterial cells were centrifuged, pelleted, and washed twice with sterile phosphate-buffered saline (PBS). The collected cells were subsequently diluted in PBS to obtain the *P*. *aeruginosa* inoculum (10^6^ CFU/mL, determine by quantitative plate read).

### Mouse air pouch model of *P*. *seruginosa* infection

Male BALB/c mice (age: 6–8 weeks and weight: 30–35 g, n = 36, 6 groups consisting 6 animals each) were procured from Adita Biosys, Tumakuru, India (CCSEA No:1868/PO/RcBt/S/16/CPCSEA) and housed in standard polycarbonate cages (Size: L 421 x B 290 x H 190 mm) under standard laboratory condition (room temperature; relative humidity: 30–70%; and under 12hr light and 12hr dark cycle). The study protocol was approved by Institutional Animal Ethics Committee (IAEC) (Proposal No. JSSAHER/CPT/IAEC/047/2022), JSS academy of higher education and research, Mysuru, Karnataka, India, following regulations of Committee for Control and supervision of experiments on animals (CCSEA), Government of India, guidelines. After one week acclimatization, the mice were randomly assigned into 6 groups (Table 2, n = 6 per group). Air pouch formation and subsequent bacterial inoculation were performed following a published protocol [14]. Briefly, 10 mL sterile air was injected into the loose dorsal tissue of each mouse and then the mouse was transferred back into the cage. One day before bacterial inoculation, 5 mL sterile air was injected into the air pouch to maintain patency. On inoculation day, each mouse was injected with 0.2 mL *P*. *aeruginosa* (10^6^ CFU/mL) into the subcutaneous air pouch. For the negative control of infection G-1 group, 0.2 mL sterile PBS was injected into the mouse air pouch. Clinical signs and symptoms were monitored and recorded daily during the acclimatization (7 days) as well as experimentation days (7 days). At the end of the treatment period (on day 7) all the animals (36 animals) were euthanized using CO_2_ euthanasia.

**Table 2:** Grouping (n = 6 per group) and treatment.

Group	Treatment	Dose
G-1	AP+ Saline (negative control)	Saline, 0.2 mL
G-2	AP+MI (positive control)	MI: 0.2 mL
G-3	AP+MI+BITC	BITC: 25 mg/kg
G-4	AP+MI+BITC	BITC: 50 mg/kg
G-5	AP+MI+GS	GS: 10 mg/kg
G-6	AP+MI+CH	CH: 10 mg/kg

AP: air pouch, MI: microbial inoculum, BITC: benzyl isothiocyanate, GS: gentamycin sulfate, and CH: ceftiofur hydrochloride.

### Determination of bacterial load in the air pouch of each treatment group

Thirty minutes after bacterial inoculation, treatment was initiated for each group (Table 2). For each type of treatment, the injection volume was 0.2 mL into the air pouch. The treatments were allowed to proceed for 7 days. Then, 3 mice from each group were randomly selected and anaesthetized using ketamine-xylazine (50mg/mg-5mg/kg). The air pouch was injected with 3 mL sterile PBS and gently massaged for 5 min before 2 mL fluid was extracted using a 26-G needle. The air pouch liquid was plated on Nutrient Agar plates. After culture, the number of bacterial colonies formed on each agar plate was counted as the bacterial load in the air pouch.

### Determination of viable and dead infiltrated cells in the air pouch

Air pouch liquid was centrifuged at 1500 RPM at 4°C for 10 min to separate the infiltrated mouse immune cells. Cell pellet was washed twice with PBS (pH 7.2) before being suspended in Dulbecco’s Modified Eagle Medium containing 5% fetal calf serum. Then, 100 μL of cells were mixed with 100 μL trypan blue solution (0.4% w/w), and the numbers of total and viable cells were measured using a hemocytometer. The number of dead cells was obtained by subtracting the number of viable cells from the number of total cells.

### Histopathological analysis of mouse air pouch skin tissue

At the end of treatment, two mice from each group were randomly selected and euthanized. Skin tissues were collected from the air pouches, processed to have section thickness of 3–5 μm, and stained by hematoxylin and eosin (H&E) stain. Histopathological analysis of the mouse air pouch skin tissues was performed by a certified veterinary pathologist at JSS Academy of Higher Education & Research (India).

## Conclusions

In the current study, we undertook a comparative *in vivo* study on the antimicrobial activity of BITC with a positive antibiotic control, gentamycin sulfate, and a negative antibiotic control, ceftiofur hydrochloride. Our results showed that BITC exhibited comparable antimicrobial activity as gentamycin sulfate but much lower level of mouse immune cell infiltration and local toxicity to mouse air pouch skin tissues, making it a promising alternative candidate to antibiotics. Further investigation, including pharmacokinetics/pharmacodynamics, clinical trial, and drug formulation studies, is warranted to confirm whether BITC could indeed be used as an alternative to antibiotics.

## Supporting information

S1 File Research data PLoS One (Fig 1: Colony count; and Fig 2: Count of viable and dead infiltrated immune cells). (XLSX)
